# Trend of some Tuberculosis Indices in Iran during 25 yr Period (1990-2014) 

**Published:** 2016-09-07

**Authors:** Salman Khazae, Erfan Ayubi, Mohammad Ali Mansournia, Hosein Rafiemanesh

**Affiliations:** ^a^ Department of Epidemiology, School of Public Health, Hamadan University of Medical Sciences, Hamadan, Iran; ^b^ School of Public Health, Zabol University of Medical Sciences, Zabol, Iran; ^c^ Department of Epidemiology & Biostatistics, School of Public Health, Tehran University of Medical Sciences, Tehran, Iran; ^d^ Students` Research Committee, School of Public Health, Shahid Beheshti University of Medical Sciences, Tehran, Iran

**Keywords:** Tuberculosis, Segmented regression, Annual Percent Change, Iran

## Abstract

**Background:** Investigation of tuberculosis (TB)-specific indices including prevalence of TB, mortality of TB cases excluding HIV, HIV/TB mortality, incidence of TB (all forms), HIV/TB incidence as well as case detection and related trends is a crucial step in evaluation of program performance and strategies success. Besides, estimating the number and time of change points for TB incidence can help to detect effective factors in TB control. Therefore, the current study aimed to determine the trend of aforementioned indices in Iran during a 25 yr period (1990 to 2014).

**Methods:** Data on trend of TB in Iran was extracted from WHO regional office reports during 1990-2014. For determining the trend of TB indices, Annual Percent Changes (APC) and Average Annual Percent Changes (AAPC) was estimated using segmented regression model.

**Results:** AAPC (95% CI) for HIV/TB mortality and HIV/TB incidence were 11.5 (9.3, 13.6) and 14.8 (13.6, 16.1), respectively, which are sign of increasing trend during the period (*P*<0.05). Other indices showed significantly decreasing trend (*P*<0.05), except for case detection rate (*P*=0.803).

**Conclusions:** The incidence, prevalence, and death rates of TB had shown a decreasing trend in general population, regarded as a useful indicator of achievements of Millennium Development Goals (MDGs) and effectiveness of interventional programs. Increasing trend of incidence and mortality of TB in HIV infected patients, needs conducting more intervention strategies in health care programs.

## Introduction

Tuberculosis (TB) has become one of the greatest human health challenges in the world especially in low-income countries so that, it is the second infectious cause of death after HIV^1,2^. According to the importance of TB in view of public health, in 1991, WHO had declared TB as an important global emergency in 1991^3,4^. A decade after introduction of Directly Observed Treatment, Short-Course ((DOTS), nearly all countries in the world have achieved to WHO 2005 goal targeting with decreasing smear-positive pulmonary cases by 70%, and treatment success rate of TB by 85% ^5^.

Despite of international attempts, conducting inefficient prevention and control programs in high burden countries still exists ^6,7^. Factors including poverty, population growth, emigration, HIV pandemic, insufficient availability to diagnostics services and treatment modalities, disproportionality of health care allocations are introduced as main causes of increasing TB cases ^6,8-12^. Due to insufficient progress in TB control programs with DOTS, other performances and activities such as DOTS II or Stop TB strategy were embedded in health care programs^3,4^. In order to operationalize the section related to tuberculosis in sixth aim from goals set of United Nations millennium development goals (MDGs), "prevalence decrease" and "death due to tuberculosis" indices are set as control program of tuberculosis by 50% in 2015 compared to 1990 ^13,14^, hence 2015 was turning point in fight against tuberculosis. Year 2015 was the last deadline of goals of MDGs and entering to a new era of sustainable development goals (SDGs) with moving from Stop TB strategy to End TB ^15^.

In line of the third MDGs, the estimated number of people with TB in recent years was decreased in the world. During 2000-2014 TB incidence rate decreased by 18%, on average 1.5% every year ^15^. Decrease in TB prevalence according to MDGs was noticeable as well. It has decreased by 50% compared to 1990, equivalent to 150 out of 300 prevalent cases and 15 out of 30 fatality cases (per 100,000 populations) ^15^. In Iran, the incidence rate of TB was decreasing, while since 1964, from 142 cases/100000 has reached to 13.7/100000 in 2013 ^16^.

Investigation of TB-specific indices and related trends is a crucial step in evaluation of program performance and strategies success. Besides, estimating the number and time of change points for TB incidence can help to detect effective factors in TB control. Therefore, the current study attempted to investigate the TB-specific indices including prevalence of TB, mortality of TB cases excluding HIV, HIV/TB mortality, incidence of TB (all forms), HIV/TB incidence and case detection in Iran during a 25 yr period (1990 to 2014).

## Methods

This cross-sectional study was performed in 2016. Since 1990, WHO routinely have collected data on the number of TB cases in member states each yr. Some methods currently used by WHO to estimate TB incidence and prevalence in countries. For example in Iran, case notification data combined with expert opinion about case detection gaps is used to estimate levels of under-reporting and under-diagnosis. Therefore, our data for trend of TB in Iran extracted from WHO regional office during 1990-2014 available from: http://www.who.int/tb/country/data/download/en/.

We examined estimated trend of all forms of TB prevalence, incidence (per 100,000 population for above indexes), case detection rate, mortality trends, estimated mortality for all forms of TB cases (excluding HIV), estimated incidence of TB cases who are HIV-positive, and case detection rate for all forms (%).

Segmented analysis was used because of non-constant trend of TB over period. This model assumes that the change in the rates is constant over each time segment called change points, but varies among different time segments ^17^. Like the least squares regression method, the Joinpoint program is used to find the best-fit line through several years of data. However, the Joinpoint program uses an algorithm that tests whether a multi-segmented line is a significantly better fit then a straight or less-segmented line.‏ Joinpoint regression analysis involves fitting a series of joined straight lines on a log scale to the trends. The aim of the approach is to identify possible Joinpoints where a significant change in the trend occurs ^18^.

The trend, Annual Percent Change (APC), and Average Annual Percent Change (AAPC) of TB were considered as independent variable whereas; prevalence, incidence, mortality, and case detection rates of TB (per 100,000 population) during 1990-2014 were as dependent ones. APC is a way to measure trends of disease over time, and AAPC is the summary of trend and determines the interval of years, to determine the summery statistics of trend.

*P*. value less than 0.05 was considered as significance level. All the statistical analyses had carried out using Joinpoint software, V3.5.1.

## Results

The maximum estimated prevalence of all forms of TB was 55 (28-90) in 1994 and was decreasing afterwards until 2010. The estimated mortality rate of all forms of TB except HIV cases had decreased to 5.5 (4-7.3) during 1994-2009, however, after that period this trend has become upward. The trend of estimated mortality of TB cases that were HIV-positive had an increasing trend in1990 to 2014. Estimated incidence of all forms had increased during 6 first years of the study and decreased in 1996 to 2008. On the other hand, estimated incidence of TB cases that were HIV-positive had been increasing from beginning to the end of the study. We found no distinct trend in case detection rates of TB throughout the study period (Table 1).

**Table 1:** Estimated rate of some Tuberculosis indices (95% CI) in Iran, 1990-2014

**Year**	**Prevalence** **(95% CI)** ^a^	**TB Mortality** **(95% CI)** ^b^	**HIV/TB Mortality** **(95% CI)** ^c^	**Incidence** **(95% CI)** ^d^	**HIV/TB Incidence** **(95% CI)** ^e^	**Case detection rate** **(95% CI)**
1990	50 (25, 82)	5.00 (3.50, 6.70)	0.01 (0.00, 0.01)	32 (28, 37)	0.05 (0.04, .070)	51 (44, 60)
1991	51 (26, 85)	5.10 (3.60, 6.80)	0.01 (0.00, 0.01)	34 (30, 38)	0.07 (0.05, 0.08)	74 (66, 83)
1992	53 (27, 88)	5.30 (3.80, 7.00)	0.01 (0.00, 0.01)	35 (31, 38)	0.08 (0.06, 0.09)	70 (63, 78)
1993	54 (28, 90)	5.50 (3.90, 7.20)	0.01 (0.00, 0.01)	35 (32, 39)	0.09 (0.07, 0.10)	99 (90, 110)
1994	55 (28, 90)	5.50 (4.00, 7.30)	0.01 (0.01, 0.01)	35 (32, 39)	0.10 (0.08, 0.13)	62 (56, 68)
1995	54 (27, 89)	5.40 (3.90, 7.20)	0.01 (0.01, 0.01)	35 (31, 39)	0.13 (0.11, 0.16)	76 (69, 84)
1996	51 (26, 85)	5.10 (3.70, 6.80)	0.01 (0.01, 0.02)	34 (30, 37)	0.18 (0.15, 0.22)	69 (62, 77)
1997	48 (24, 80)	4.80 (3.50, 6.40)	0.01 (0.01, 0.02)	32 (29, 35)	0.26 (0.21, 0.32)	64 (58, 71)
1998	45 (23, 75)	4.60 (3.30, 6.10)	0.02 (0.01, 0.02)	30 (27, 33)	0.38 (0.31, 0.46)	63 (56, 70)
1999	42 (21, 70)	4.30 (3.00, 5.70)	0.02 (0.01, 0.03)	27 (24, 31)	0.54 (0.44, 0.65)	68 (60, 77)
2000	39 (20, 64)	3.80 (2.70, 5.10)	0.02 (0.02, 0.03)	26 (22, 29)	0.73 (0.59, 0.88)	70 (62, 81)
2001	36 (18, 60)	3.50 (2.50, 4.70)	0.03 (0.02, 0.04)	24 (21, 27)	0.92 (0.75, 1.10)	73 (65, 84)
2002	34 (17, 57)	3.30 (2.30, 4.40)	0.03 (0.02, 0.04)	23 (20, 26)	1.10 (0.90, 1.10)	74 (66, 84)
2003	33 (17, 54)	3.20 (2.30, 4.20)	0.04 (0.03, 0.05)	22 (19, 24)	1.30 (1.00, 1.50)	73 (66, 82)
2004	32 (16, 53)	3.20 (2.30, 4.20)	0.05 (0.03, 0.07)	21 (19, 23)	1.40 (1.20, 1.70)	70 (62, 78)
2005	31 (16, 51)	3.10 (2.20, 4.10)	0.06 (0.04, 0.08)	20 (18, 23)	1.60 (1.30, 1.90)	64 (58, 72)
2006	30 (16, 50)	3.10 (2.20, 4.00)	0.06 (0.04, 0.09)	20 (18, 22)	1.70 (1.40, 2.00)	66 (60, 73)
2007	30 (15, 49)	3.00 (2.2, 4.00)	0.07 (0.05, 0.10)	20 (18, 21)	1.80 (1.40, 2.10)	66 (61, 72)
2008	30 (15, 49)	3.00 (2.10, 4.00)	0.08 (0.05, 0.10)	19 (18, 22)	1.90 (1.50, 2.30)	67 (61, 74)
2009	30 (15, 49)	3.00 (2.10, 4.00)	0.09 (0.06, 0.12)	20 (17, 22)	2.00 (1.60, 2.40)	71 (62, 81)
2010	30 (15, 50)	3.10 (2.20, 4.10)	0.09 (0.06, 0.13)	20 (17, 23)	2.10 (1.70, 2.50)	71 (60, 84)
2011	31 (16, 51)	3.20 (2.20, 4.30)	0.10 (0.07, 0.14)	20 (17, 24)	2.10 (1.70, 2.60)	73 (61, 88)
2012	32 (16, 52)	3.30 (2.30, 4.50)	0.11 (0.07, 0.16)	21 (17, 25)	2.20 (1.80, 2.70)	70 (59, 86)
2013	33 (17, 54)	3.40 (2.30, 4.60)	0.12(0.08, 0.17)	21 (17, 25)	2.30 (1.90, 2.80)	68 (56, 84)
2014	33 (17, 55)	3.50 (2.40, 4.70)	0.14(0.09, 0.19)	22 (18, 26)	2.40 (1.90, 2.80)	60 (50, 74)

a) Estimated prevalence of TB (all forms) per 100,000 population; (b) Estimated mortality of TB cases (all forms, excluding HIV) per 100,000 population; (c) Estimated mortality of TB cases who are HIV-positive, per 100,000 population; (d) Estimated incidence (all forms) per 100,000 population; (e) Estimated incidence of TB cases who are HIV-positive per 100,000 population

**Figure 1:**
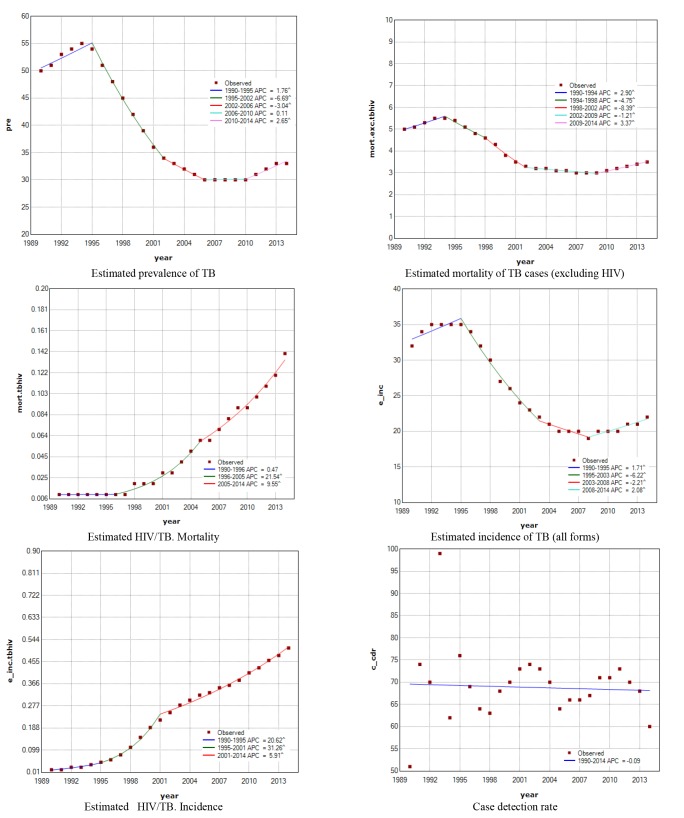
Trend and Annual Percent change (APC) for TB indices in Iran, 1990-2014

The number and locale of change point (s) for each index was ranged from 0 to 4 (Table 2). AAPC for these indices estimated HIV/TB mortality (11.5 (95% CI: 9.3, 13.6)), and estimated HIV/TB incidence (14.8 (95% CI: 13.6, 16.1)) which have a positive sign indicating increasing trend during that period (*P* <0.05). For other indicators, the trend was significantly downward (*P* <0.05), except for case detection rate (*P* =0.803). The maximum estimated AAPC was attributed to the estimated HIV/TB incidence (AAPC=14.8) and the minimum value was attributed to estimated prevalence of TB and estimated incidence of TB (all forms) (AAPC=-1.7). Although we observed an increasing trend for all the indices of interest that fluctuated from 2.1 (for estimated incidence of TB) to 9.5 (for Estimated HIV/TB mortality) according to last segment and based on APC. The results of APC and estimated trends for all TB indices are further shown in Figure 1.

**Table 2:** Number and local of change points, APC and AAPC for TB indices in Iran, 1990-2014

**Years, No. of change points**	**APC (95% CI)**	**AAPC (95% CI)**	***P*** ** value**
**Estimated prevalence of TB, 4**		-1.70 (-2.00, -1.40)	<0.05
1990-1995	1.80 (1.20, 2.30)		
1995-2002	-6.70 (-7.10, -6.30)		
2002-2006	-3.00 (-4.20, -1.90)		
2006-2010	0.10 (-1.10, 1.30)		
2010-2014	2.60 (1.90, 3.40)		
**Estimated mortality of TB cases (excluding HIV), 4**		-1.50 (-1.10, -1.80)	<0.05
1990-1994	2.90 (2.10, 3.70)		
1994-1998	-4.70 (-6.00, -3.50)		
1998-2002	-8.40 (-9.60, -7.20)		
2002-2009	-1.20 (-1.60, -0.80)		
2009-2014	3.40 (2.80, 4.00)		
**Estimated HIV/TB Mortality, 2**		11.50 (9.30, 13.60)	<0.05
1990-1996	0.50 (-4.60, 5.80)		
1996-2005	21.50 (17.60, 25.60)		
2005-2014	9.50 (6.50, 12.60)		
**Estimated incidence of TB (all forms), 3**		-1.70 (-2.20, -1.20)	<0.05
1190-1995	1.70 (0.40, 3.00)		
1995-2003	-6.20 (-6.90, -5.50)		
2003-2008	-2.20 (-3.90, -0.40)		
2008-2014	2.10 (1.10, 3.10)		
**Estimated HIV/TB Incidence, 2**		14.80 (13.60, 16.10)	<0.05
1990-1995	20.60 (16.70, 24.70)		
1995-2001	31.30 (26.90, 35.70)		
2001,2014	5.90 (5.10, 6.70)		
**Case detection rate, 0**		-0.10 (-0.70, 0.60)	0.8
1990-2014	-0.10 (-0.70, 0.60)		

## Discussion

Results of the present study showed that the trend of prevalence and death rate due to all forms of TB in Iran had decreased in 1994 to 2010 and increased afterwards. While incidence rate revealed a curvy trend starting with increasing trend in time period of 1990-1995, decreasing trend until 2008, and increasing trend from 2008 to 2014.

Our results revealed that there were some incomplete achievements in the third MDGs in Iran regarding TB. According to these goals, prevalence and death due to TB in 2015 decreased to 50% compared to 1990 that in Iran prevalence reached to 33 cases from 50 cases (100 thousand people) and death rate to 3.5 cases from 5 (100000 people). However, according to WHO report in 2015 these goals partly have achieved. Based on the report from six regions of WHO, only Africa and Europe regions have not met the 50% goal for death reduction; while Africa, Eastern Mediterranean, and Europe have not met the 50% goal for prevalence reduction ^15^.

Current study used regression models and Joint Point software to study the trend of incidence rate, prevalence, and death rates in all TB cases and HIV positives separately. One of main advantages of this method compared to other common methods of trend investigation is to diagnose and specify trend change points and consequently present distinct processes for a period of time ^18^. Therefore, study of TB disease process for a long period of 25 yr in this study is certainly influenced by interventions and various events throughout this period such as DOTS treatment programs, or emergence of AIDS epidemic that can justify disease trend partly or in a whole.

Death rate trend of TB for Egypt during 1992-2011 showed obvious reduction^19^. Moreover, Ohmori et.al showed reduction of death rate of tuberculosis was during 1910 to 1990 for Japan, France, United States and The Netherlands ^20^. In one Study by Kazemnejad et al. on trend of TB incidence and death rates around the world found that the AAPC for incidence and death rate of TB had a reduction trend in the world as well as in all regions of WHO during 2001-2010 ^21^, as endorsed in our results in a country of Eastern Mediterranean region. Additionally, Kazemnejad et.al. found a significant increase trend in TB case detection rate for the world and for five WHO regions ^21^. We found that death and incidence rates of TB in people with HIV were increasing for period of the study. In this context, the highest increase was in 1995 until 2001 for incidence rate and in 1996-2005 for death rate. In Addition, we found no significant trends in terms of case detection rate in the whole study period. Saad-Hussein et.al in Egypt also reported no particular trend in case detection rate for 1996 to 2012 ^19^. Ibrahim Al-Orainey et.al evaluated tuberculosis incidence trends in Saudi Arabia over 1991-2010. In that study, TB trend was rising over the first 10 yr of the study period then it started to fall slightly. The incidence increased with age, but only people older than 45 yr showed a declining trend ^22^.

Joint Point analysis showed 5 various time process for TB prevalence. There was increased prevalence before 1995, and two separate reductions before 2006 attributed to the start of present treatment programs by WHO and being successful in these programs. TB prevalence had constant trend within 2006 to 2010 and then increased. This increment trend can be attributed to the emergence of inappropriate and incomplete treatments, emergence of drug-resistant tuberculosis, emergence of tuberculosis occurrence in people with HIV, and start of AIDS epidemic ^23^.We found that there was an increasing trend for incidence and death of TB in people with HIV in the whole study period. The highest increase for incidence occurred in 1995-2001, while the highest increase for death occurred in 1996-2005.

A decreasing trend in registered TB cases was reported by Tamami et.al in Fiji^24^. Alongside with other countries such as Fiji, this decrease was partly due to different interventions such as introduction of BCG vaccination, start of Primary Health Cares (PHC), and presence of lavatory and radiography facilitations ^24^.

Investigation of TB trend by Rahimi et.al in West Azerbaijan Province also reported a decreasing incidence before 2003 followed by a mild increase with steepest slope for 2008 ^25^. This is consistent with our results, which showed increasing trend for 2008-2014 following decreased one.

There is a growing trend of TB-HIV comorbidity all around the world, which makes TB, and HIV strongly interrelated. The most common opportunistic infection and cause of death in people with HIV worldwide is TB ^1,2,19^. HIV infection can affect incidence and death of TB via different pathways including degradation of TB treatment, increasing probability of TB relapse and, on-treatment side effects or deaths, and increasing probability of TB transmission^26^. Despite the reports of other authors in Iran indicating no significant occurrence of TB-HIV comorbidity, the increasing trend of TB during 1995-2005 can be proportionally attributed to the progressive epidemic of AIDS^25^.

One limitation of this study was that because lack of access to data, changes in trend of some indices of TB in separate provinces was not assessed. In addition, examination the effect of related factors on the TB incidence such as ethnic groups, geographical regions and individual’s socio-economic status was not assessed. It is recommended that these issues considered in future studies

## Conclusions

The incidence, prevalence, and death rates of TB had shown a decreasing trend regarded as a useful indicator of achievements of MDGs as well as effectiveness of interventional programs. Ignorance of increasing trend of incidence and death of TB in people with HIV, however, may lead to a tremendous consequence beyond the studies indices.

## Acknowledgments

We would like to gratefully and sincerely thank Dr. Manoochahr Karami for his recommendations about the statistical matter of joint point software.

## Conflict of interest statement

The authors declare that there is no conflict of interests.

## Highlights

- HIV/TB mortality and incidence rates have increasing trend in Iran
- incidence, prevalence, and death rates of TB have decreasing trend in Iran
- The average annual of HIV/TB mortality and incidence have increased more than 10%
- Approximately after 2010, decreasing trend of incidence, prevalence, and death rates of TB has stopped and its direction has changed.
- The time pattern of TB incidence and prevalence were relatively unsteady in Iran from 1990 to 2014
